# JMSC: Joint Spatial–Temporal Modeling with Semantic Completion for Audio–Visual Learning

**DOI:** 10.3390/s26041288

**Published:** 2026-02-16

**Authors:** Xinfu Xu, Fan Yang, Zhibin Yu

**Affiliations:** 1 Faculty of Information Science and Engineering, Ocean University of China, Qingdao 266100, China; xuxinfu@stu.ouc.edu.cn (X.X.); yf6278@stu.ouc.edu.cn (F.Y.); 2 Key Laboratory of Ocean Observation and Information of Hainan Province, Sanya 572000, China

**Keywords:** multimodal fusion, audio–visual learning, deep learning

## Abstract

Audio–visual learning seeks to achieve holistic scene understanding by integrating auditory and visual cues. Early research focused on fully fine-tuning pre-trained models, incurring high computational costs. Consequently, recent studies have adopted parameter-efficient tuning methods to adapt large-scale vision models to the audio–visual domain. Despite the competitive performance of existing methods, several challenges persist. Firstly, effectively leveraging the complementary semantics between the audio and visual modalities remains difficult, as these two modalities capture fundamentally different aspects of a video. Secondly, comprehending dynamic video context is challenging because both spatial attributes (such as scale) and temporal characteristics (such as motion) of objects co-evolve over time, making semantic comprehension more complex. To address these challenges, we propose a novel framework, named Joint Spatial–Temporal Modeling with Semantic Completion (JMSC). JMSC introduces cross-modal latent reconstruction, which moves beyond shallow correlation by encouraging the model to reconstruct one modality’s complete semantic summary from a masked version of its counterpart. Furthermore, JMSC learns a unified representation of video spatial attributes and temporal changes by jointly modeling them under audio guidance, enabling accurate localization and consistent tracking in dynamic video scenes. Experimental results demonstrate that JMSC achieves state-of-the-art performance across multiple downstream tasks while maintaining high computational efficiency.

## 1. Introduction

Human perception fundamentally relies on sight and hearing [1,2], which together capture and process a diverse spectrum of external information. This foundational principle has driven significant research efforts to explore audio–visual learning [3,4,5,6,7,8], aiming to develop intelligent systems capable of emulating human-like multimodal perception.

In the realm of audio–visual learning, the deployment of large-scale pre-trained models has yielded remarkable outcomes. While full fine-tuning of such models has achieved promising results [9,10,11,12], it comes with substantial memory usage and extensive trainable parameters. Recent studies have thus explored parameter-efficient transfer techniques, such as prompt tuning and adapters, to efficiently transfer large-scale vision models to the audio–visual domain [13,14]. This approach is promising for the following two reasons. First, it alleviates the burden of retraining the model from scratch. Second, it provides a strong foundation for cross-modal learning, tasking the model to discover and leverage the intrinsic correlations between the naturally complementary audio and visual signals.

Despite the significant progress previous work has made in fundamental tasks like audio–visual classification and retrieval, challenges persist in advanced audio–visual scenarios. In particular, we argue that current approaches still lack principled solutions to two essential research questions (RQs):

RQ1: How to leverage the complementary semantics between audio and visual modalities effectively? Existing methods, such as CAV-MAE [15] and AVAF-NET [16], have made progress by employing contrastive learning to establish global correspondences between audio and visual features. While this captures coarse co-occurrences, it often overlooks the fine-grained complementary information needed for deeper semantic understanding. For example, in a scene where a person is speaking next to a silent dog, global alignment tends to associate all present objects with the sound. This captures co-occurrence but may not clearly distinguish the true origin of the sound from a contextual bystander.

**Figure 1:**
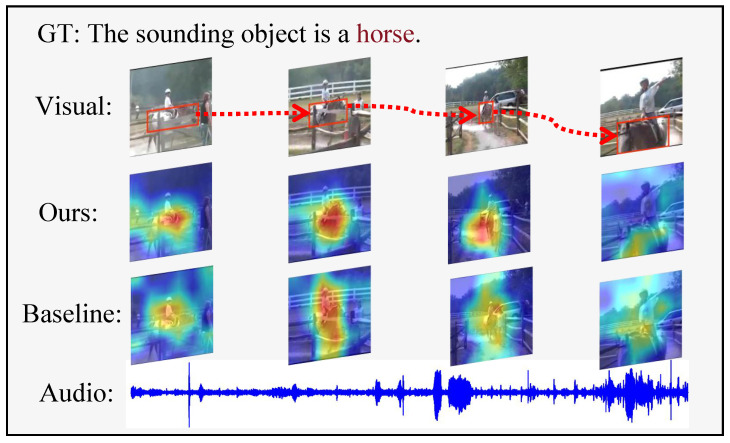
Heatmap on audio event localization using Grad-CAM [18]. It can be observed that the main object’s size and position constantly change, leading to suboptimal results for the baseline using only adapters to capture temporal cues. In contrast, JMSC, by joint integration of temporal and spatial features, is capable of precisely locating the sounding object.

RQ2: How to jointly model the co-evolution of spatial and temporal features? As shown in Figure 1, capturing these co-changes is challenging because the model must simultaneously preserve spatial details for localization and encode long-range temporal dependencies. Prior studies [14,17] either focus on capturing temporal variations or separately model spatial and temporal features, thereby neglecting their co-evolution and leading to suboptimal performance in dynamic scenes. This observation motivates us to design a model with a unified spatial–temporal representation that can explicitly model their interaction.

**Figure 2:**
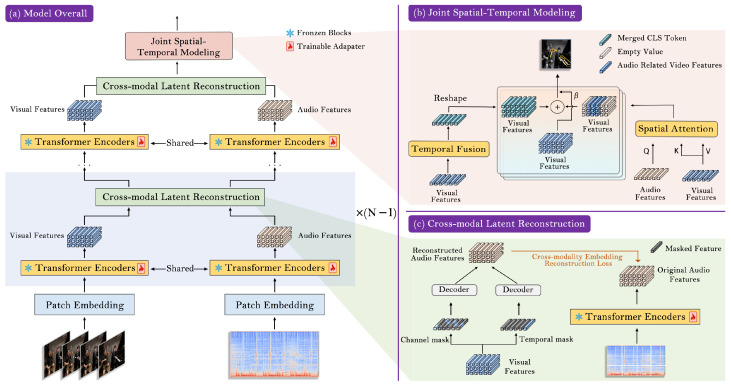
Overview of the proposed JMSC. (**a**) Overall pipeline, which employs cross-modal latent reconstruction after each encoder block, and the joint spatial–temporal modeling is applied only in the second-to-last layer. (**b**) For joint spatial–temporal modeling, we combine video features activated by audio with temporally fused CLS tokens. (**c**) For cross-modal latent reconstruction, we utilize the masked modality features to reconstruct the complete features of the counterpart modality. We take the visual modality as an example to illustrate (**c**), and the same applies to the audio modality.

To answer these questions, we propose JMSC, a novel framework that achieves strong audio–visual learning under the parameter-efficient paradigm. As illustrated in Figure 2, our approach introduces cross-modal latent reconstruction (CMLR), which operates at the shared latent representation level rather than the raw feature space. Specifically, we selectively mask embeddings of one modality along either the temporal or channel dimension and require the model to reconstruct the corresponding representation of the counterpart modality. This design encourages the model to infer missing semantic cues across modalities and capture deeper cross-modal dependencies. In addition, JMSC introduces joint spatial–temporal modeling (JSTM) to capture how an object’s spatial attributes and temporal state evolve jointly. It uniquely combines an audio-guided mechanism for precise spatial localization with a temporal frame-level fusion strategy to capture temporal dependencies. By fusing these two specialized representations, our model builds a cohesive understanding of video content, ensuring that localized objects are consistently tracked over time. In summary, our key contributions are as follows:

- We propose a cross-modal latent reconstruction objective that deviates from alignment-driven paradigms and instead casts cross-modal interaction as a generative semantic inference problem, effectively bridging the audio–visual representation gap and better exploiting complementary cues.
- We introduce a novel joint spatial–temporal modeling strategy that models how objects’ locations and motions change together over time, yielding precise localization and consistent tracking of sounding objects in dynamic video scenes.
- Extensive experiments are conducted on various tasks to demonstrate the superiority and generalization of our model. JMSC achieves state-of-the-art performance with high computational efficiency.

## 2. Related Works

### 2.1. Audio–Visual Learning

Audio–visual learning aims to explore the association and complementarity between audio and visual modalities to better perceive audio–visual scenarios. By integrating audio and visual inputs, audio–visual learning enhances model performance and improves generalization capabilities. This area includes a wide range of applications, such as audio–visual event localization, audio–visual segmentation, and audio–visual question answering. For instance, audio–visual event localization aims to detect events occurring simultaneously in both visual and audio streams within a video [19,20,21]. Audio–visual segmentation performs pixel-level localization of sound-emitting objects [22,23,24]. Additionally, audio–visual question answering is an emerging task that integrates visual and auditory information to answer questions based on their associations [25,26]. Early research primarily focused on employing attention mechanisms to capture multimodal relations. For instance, CMRAN [27] proposed a cross-modal attention module to extract interactive information from audio–visual pairs. To mitigate the interference of background noise, CMBS [28] introduced time-level and event-level background suppression mechanisms. Distinct from attention-based approaches, recent studies have shifted to enhance cross-modal alignment. PSP [20] introduced a positive sample method to filter out low-correlation audio–visual pairs. Building on this, CPSP [29] further incorporated segment-level and video-level contrastive learning strategies. Similarly, AVAF-Net [16] utilized temporal and spatial contrastive learning to align modalities effectively.

However, while previous approaches leverage contrastive learning to establish correspondences, they often neglect a comprehensive understanding of the semantic relationships between modalities. To address this limitation, we introduce cross-modal latent reconstruction, which fosters a more robust and deep understanding of audio–visual interactions.

### 2.2. Parameter-Efficient Transfer Learning

Parameter-Efficient Transfer Learning (PETL) adapts pre-trained foundation models to diverse tasks by fine-tuning only newly introduced parameters while keeping the original model weights frozen. Generally, PETL technologies can be categorized into methods like Adapter [30,31,32], Prompt [14,33], and LoRA [34,35]. Adapter-based approaches introduce additional lightweight branches to adapt large pre-trained models to downstream tasks. Prompt-based methods customize the model for specific tasks by concatenating learnable prompts with the input, whereas LoRA focuses on learning low-rank factorizations of the model weights. Currently, parameter-efficient transfer learning in multimodal learning has been predominantly explored in the vision–language domain [36,37], with relatively limited investigation in the audio–visual field. Research on PETL in the audio–visual domain remains relatively underexplored and has only recently begun to gain attention. LAVISH [38] first employed specifically designed inserted adapters to facilitate the interaction between audio and visual features, but neglected the spatial–temporal adaptation of modality-specific signals. DG-SCT [13] introduced a dual-guidance attention mechanism. They utilize one modality to guide the feature extraction of the counterpart modality across spatial, channel, and temporal dimensions. However, the specifically designed attention mechanisms unavoidably introduce a substantial number of trainable parameters. CoPL [14] utilizes task-specific prompts to achieve modal-specific fine-tuning, but simple prompts struggle to capture the complex dynamics of videos. STG-CMA [17] further proposed a spatiotemporal-global cross-modal adaptation module that employs two bidirectional audio–visual adapters to model the cross-modal dependencies; however, the exclusive use of adapters struggles to capture the coordinated evolution of temporal and spatial features, as it lacks a dedicated mechanism to model their interdependent dynamics.

Unlike these methods, which rely on adapters or prompts to integrate spatial and temporal features, making it challenging to capture the dynamic changes in videos effectively. Our model introduces a novel audio-guided spatial–temporal feature interaction strategy that dynamically highlights active visual regions and maintains temporal consistency, thereby achieving precise localization and robust tracking of sound sources.

## 3. Nomenclature

To enhance the readability of the mathematical notations used in this paper, we provide a summary of the nomenclature and symbols in Table 1.

## 4. Methods

Our objective is to establish a new paradigm of parameter-efficient audio–visual learning, which addresses the challenge of effectively leveraging multimodal information and models spatio-temporal dependencies in a unified way. Previous studies have demonstrated the effectiveness of ImageNet-pre-trained vision encoders in capturing audio features [13,17,38]. Motivated by these findings, we adopt a shared encoder pre-trained on ImageNet to process multimodal information for better efficiency. We utilize SwinTransformerV2 (Swin-V2-L) [39] and Vision Transformer-Large (ViT-L-16) [40] as both the vision and audio encoders. The following section provides a detailed explanation of our proposed approach.

We begin by introducing the multimodal input in Section 4.1, followed by our adapter design in Section 4.2. Next, Section 4.3 presents the joint spatial–temporal feature interaction module, which enhances video content understanding. In Section 4.4, we introduce the cross-modal latent reconstruction strategy that emphasizes fine-grained cross-modal correspondence. Finally, Section 4.5 describes the overall training objective, including the proposed loss functions used to optimize the model. Detailed explanations of each component are provided in the subsequent sections.

### 4.1. Visual and Audio Input

Visual Representation. Given a video input, we sample it into fixed frames, obtaining an RGB video frame sequence ${\left\{V_{t}\right\}}_{t=1}^{T}\in R^{T\times H\times W\times 3}$, where *T* represents the video length. The RGB frames are then projected into non-overlapping patches, followed by ViTs with an appended CLS token, producing the visual feature $v_{t}\in R^{T\times (N_{v}+1)\times D}$, where $N_{v}$ denotes the number of visual patches.

Audio Representation. Given an input audio track, we extract its Mel spectrogram ${\left\{A_{t}\right\}}_{t=1}^{T}$, where $A_{t}\in R^{L\times F}$ with time *L* and frequency bins *F*, the spectrogram is then duplicated three times to match the input format for the ViT. Similar to the visual modality, the audio spectrogram is converted into the audio feature $a_{t}\in R^{T\times (N_{a}+1)\times D}$, where $N_{a}$ is the number of audio patches.

### 4.2. Adding Adapter to Frozen Encoder

**Figure 3:**
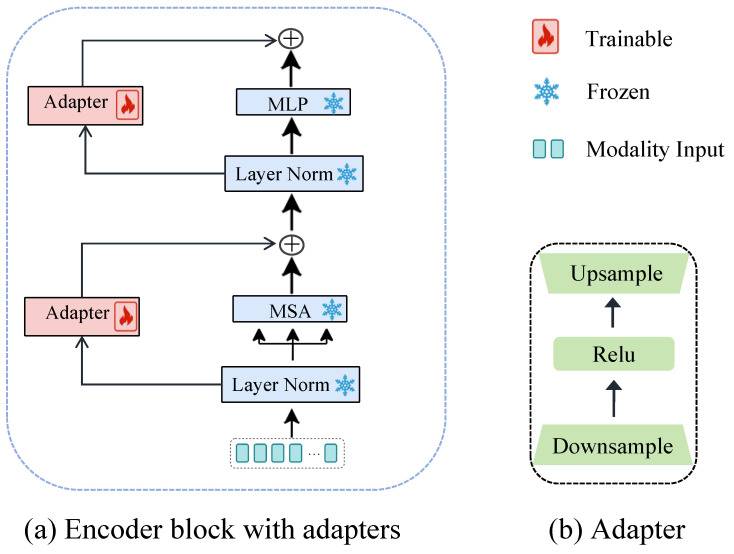
Architecture of the proposed adapter. (**a**) Illustration of how the adapter is inserted into each Transformer block in a parallel manner, where MSA denotes Multi-Head Self-Attention and MLP denotes Multi-Layer Perceptron. (**b**) Detailed structure of the adapter module.

The adapter adopts a bottleneck architecture comprising a down-projection layer $\theta_{\mathrm{down}}$, a ReLU activation function δ, and an up-projection layer $\theta_{\mathrm{up}}$, as shown in Figure 3. To mitigate the high computational cost of gradient backpropagation in a sequential setup, we integrate it in parallel with the frozen encoder block. For simplicity, we omit normalization, and the operations in each layer can be expressed as:

(1) $$\mathrm{Adapter}\left(x\right)=\theta_{\mathrm{up}}\left(\delta \left(\theta_{\mathrm{down}}\left(x\right)\right)\right)$$

(2) $${H^{\prime }}_{X}^{\left(l\right)}=\mathrm{Adapter}\left(H_{X}^{\left(l\right)}\right)+\mathrm{MSA}\left(H_{X}^{\left(l\right)}\right),$$

(3) $$H_{X}^{\left(l+1\right)}=\mathrm{Adapter}\left({H^{\prime }}_{X}^{\left(l\right)}\right)+\mathrm{MLP}\left({H^{\prime }}_{X}^{\left(l\right)}\right),$$

where $H_{X}^{\left(l\right)}$ represents the output of the *l*-th layer, and ${H^{\prime }}_{X}^{\left(l\right)}$ denotes the intermediate feature of the *l*-th layer after MHA fusion.

### 4.3. Joint Spatial–Temporal Modeling

In audio–visual downstream tasks, it is essential to explore both the dynamic spatial information within individual frames and the temporal dependencies within video clips. Unlike previous methods that treat spatial attention and temporal aggregation as separate, sequential steps, JMSC explicitly models the co-evolution of these dimensions. To achieve this, we propose joint spatial–temporal modeling. As illustrated in Figure 2b, our approach leverages audio cues to generate spatial attention maps dynamically, guiding the model to focus on audio-relevant regions in each frame. Concurrently, we aggregate these global features over time to ensure temporal consistency. Finally, we integrate the refined spatial–temporal features to generate a comprehensive video representation embodying a deep spatial–temporal understanding.

**Audio-Related Spatial Features.** The core intuition is that sound-producing events provide strong cues for localizing important visual objects. To realize this, we introduce cross-modal spatial attention that uses the audio feature as a query to attend to different spatial regions of the visual frames. Given the audio features $a_{\mathrm{cls}}\in R^{T\times D}$ and visual patch features $v_{p}\in R^{(N_{v}\cdot T)\times D}$, we perform audio-guided spatial attention in a frame-wise manner. Specifically, at each time step *t*, the audio embedding $a_{\mathrm{cls}}^{t}\in R^{D}$ and the corresponding visual patch features $v_{p}^{t}\in R^{N_{v}\times D}$ are first projected into a shared hidden space of dimension d=512 using linear projections $\Theta_{a}^{s}$ and $\Theta_{v}^{s}$, respectively. The projected audio feature is broadcast along the spatial dimension and combined with visual features via element-wise multiplication (denoted as ⊙) to obtain the audio-guided spatial representation:

(4) $$v_{t}^{\mathrm{sa}}=\Theta_{a}^{s}\left(a_{\mathrm{cls}}^{t}\right)⊙\Theta_{v}^{s}\left(v_{p}^{t}\right),\;v_{t}^{\mathrm{sa}}\in R^{N_{v}\times d}$$

To generate spatial attention weights, we apply a linear projection $\Theta^{s}$ (mapping dimension *d* to 1), followed by a sigmoid activation σ(·), yielding the audio-guided spatial attention map:

(5) $$M_{t}^{\mathrm{sa}}=\sigma \left(\Theta^{s}\left(v_{t}^{\mathrm{sa}}\right)\right)\in R^{N_{v}\times 1}$$

The visual patch features are then updated under the guidance of audio cues through a residual modulation mechanism:

(6) $$\begin{array}{c} v_{p}^{\prime t}=v_{p}^{t}⊙\left(\lambda M_{t}^{\mathrm{sa}}+1\right),v_{p}^{\prime t}\in R^{N_{v}\times D} \end{array}$$

where λ is a learnable scaling parameter initialized to 1.

To further suppress visually irrelevant regions that are weakly correlated with the audio signal, we introduce a predefined hyperparameter threshold $T_{k}$ to perform spatial filtering. Since $M_{t}^{\mathrm{sa}}$ explicitly represents the relevance of each visual region to the audio, we generate a region-level binary mask based on these attention scores. The final audio-aware visual representation is obtained as:

(7) $$\begin{array}{c} v_{k}^{t}=I\left(M_{t}^{\mathrm{sa}}>T_{k}\right)⊙v_{p}^{\prime t},v_{k}^{t}\in R^{N_{v}\times D} \end{array}$$

where I(·) denotes the indicator function. Note that the binary mask derived from $M_{t}^{\mathrm{sa}}\in R^{N_{v}\times 1}$ is broadcast along the channel dimension *D*. This operation strictly filters out non-audible visual regions while preserving the complete semantic features of the active ones.

**Fusing Temporal Features.** After refining the features spatially for each frame, we model the temporal dependencies. We adopt a simple yet effective mean pooling strategy over the sequence of CLS tokens to create a robust representation that captures temporal changes. The process is defined as:

(8) $$\begin{array}{c} v_{\mathrm{global}}^{\prime }=\frac{1}{T}\sum_{t=1}^{T}v_{t}^{\mathrm{cls}},v_{\mathrm{global}}^{\prime }\in R^{N_{v}\times 1} \end{array}$$

where $v_{t}^{\mathrm{cls}}$ denotes the visual CLS token at time *t* and *T* represents the number of frames. To integrate the global temporal context $v_{\mathrm{global}}^{\prime }$ into our spatially-refined patch features $v_{k}^{t}$, we employ a learnable fusion strategy. Specifically, the global temporal context is first broadcast to match the spatial dimensions of the patch features. Subsequently, they are concatenated and processed through a fusion module $F:R^{D}\to R^{D}$ (implemented as a linear projection layer) to generate the fused representation $v_{f}^{t}$:

(9) $$\begin{array}{c} v_{f}^{t}=F\left(\mathrm{Concat}(v_{k}^{t},\mathrm{Expand}\left(v_{\mathrm{global}}^{\prime }\right))\right)\in R^{N_{v}\times D} \end{array}$$

where Expand(·) denotes repeating the global feature vector along the spatial dimension, Concat(·) represents concatenation along the channel dimension. This allows the model to adaptively recalibrate spatial features under the guidance of temporal semantics. Finally, we introduce a residual connection between the original feature $v_{p}^{t}$ and the fused feature $v_{f}^{t}$, yielding the final enhanced representation $V_{t}^{f}$, which is subsequently fed into the final encoder layer:

(10) $$V_{t}^{f}=v_{p}^{t}+\beta v_{f}^{t},$$

where β is a learnable scalar parameter initialized to 1. By adjusting β, the network can adaptively determine the fusion ratio, leading to more robust outcomes.

### 4.4. Cross-Modal Latent Reconstruction

**Figure 4:**
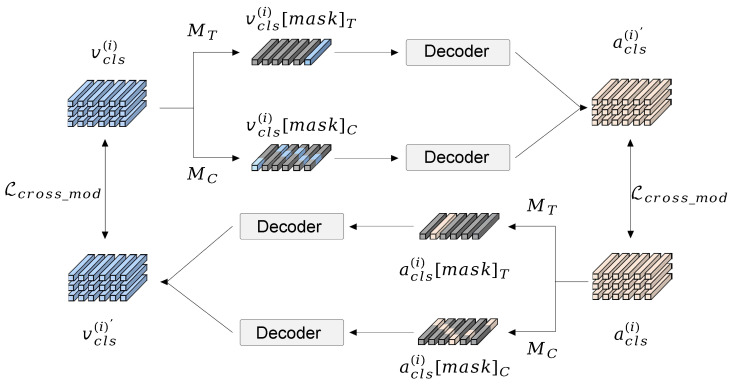
The architecture diagram of the CMLR model.

To effectively leverage the complementary semantics between modalities, we propose cross-modal latent reconstruction, which operates in the latent semantic space defined by high-level CLS tokens from each modality. As illustrated in Figure 4, CMLR takes a partially masked semantic summary of one modality from either the temporal or channel dimension and uses it to fully reconstruct the original, unmasked summary of its counterpart. This dual-objective approach fosters a deeper and more structured cross-modal understanding: it not only forces a temporal alignment of events, but also a correspondence between their underlying semantic attributes. Importantly, the reconstruction process is used only during training, incurring no additional computational cost during inference. Crucially, this design allows us to cast cross-modal interaction as a generative semantic inference problem, moving beyond the shallow contrastive learning paradigms used in prior audio–visual works.

**Latent-Space Masking Strategy.** Let $a_{\mathrm{cls}}^{\left(i\right)},v_{\mathrm{cls}}^{\left(i\right)}\in R^{T\times D}$ denote the audio and visual CLS token sequences extracted from the *i*-th encoder layer, where *T* and *D* represent the temporal length and channel dimension, respectively. We design a dual-dimensional masking strategy that independently perturbs the representations along the temporal and channel axes. Specifically, two types of binary masks are defined:

(11) $$M_{T}\in {\left\{0,1\right\}}^{T\times 1},\;M_{C}\in {\left\{0,1\right\}}^{1\times D},$$

which correspond to temporal masking and channel masking, respectively. Following the common practice in masked autoencoders (MAE) [41], a high masking ratio of 75% is adopted. Distinct from traditional MAE, which masks raw input patches (potentially destroying ground-truth structures required for dense prediction tasks like AVS), our strategy operates strictly in the latent space. Importantly, the masking operation is implemented via element-wise multiplication, such that the dimensionality of the latent representations remains unchanged. The masked representations are obtained as:

(12) $$\begin{array}{cccc} a_{\mathrm{cls}}^{\left(i\right)}{\left[\mathrm{mask}\right]}_{T} & =M_{T}⊙a_{\mathrm{cls}}^{\left(i\right)},\;\; & \;\;a_{\mathrm{cls}}^{\left(i\right)}{\left[\mathrm{mask}\right]}_{C} & =a_{\mathrm{cls}}^{\left(i\right)}⊙M_{C}, \end{array}$$

(13) $$\begin{array}{cccc} v_{\mathrm{cls}}^{\left(i\right)}{\left[\mathrm{mask}\right]}_{T} & =M_{T}⊙v_{\mathrm{cls}}^{\left(i\right)},\;\; & \;\;v_{\mathrm{cls}}^{\left(i\right)}{\left[\mathrm{mask}\right]}_{C} & =v_{\mathrm{cls}}^{\left(i\right)}⊙M_{C}, \end{array}$$

By enforcing reconstruction from these two distinct masked views, our method moves beyond shallow associations and achieves two complementary objectives:

- **Channel Masking** occludes specific semantic factors. This forces the model to infer abstract attributes (e.g., visual texture from auditory timbre) across modalities, correlating their underlying semantic components.
- **Temporal Masking** erases certain moments in time. This compels the model to learn the predictive dependency of events by understanding the temporal context provided by the other modality.

For notational simplicity, we unify both masking strategies using a generic binary mask $M\in {\left\{0,1\right\}}^{T\times D}$, which can be instantiated from either the temporal mask $M_{T}$ or the channel mask $M_{C}$ via broadcasting. Accordingly, the masked latent representation is denoted as:

(14) $$x_{\mathrm{cls}}^{\left(i\right)}\left[\mathrm{mask}\right]=M⊙x_{\mathrm{cls}}^{\left(i\right)},\;x\in \left\{a,v\right\}.$$

**Cross-modality Embedding Reconstruction.** To promote latent-level perception, the masked features from both modalities are processed through a shared decoder to reconstruct the original, unmasked representation of the other modality. Structurally, the decoder consists of a four-headed self-attention layer and a feed-forward network (FFN) implemented as two linear layers with a 4× expansion and contraction, connected by a non-linear activation function. Given the masked features, the cross-modal reconstruction process is formulated as:

(15) $$a_{\mathrm{cls}}^{{\left(i\right)}^{\prime }}=\mathrm{LN}\left(\mathrm{FFN}\left(\mathrm{SA}(v_{\mathrm{cls}}^{\left(i\right)}\left[\mathrm{mask}\right])\right)\right),$$

(16) $$v_{\mathrm{cls}}^{{\left(i\right)}^{\prime }}=\mathrm{LN}\left(\mathrm{FFN}\left(\mathrm{SA}(a_{\mathrm{cls}}^{\left(i\right)}\left[\mathrm{mask}\right])\right)\right),$$

where SA(·) denotes the self-attention operation, and LN(·) indicates Layer Normalization. $a_{\mathrm{cls}}^{{\left(i\right)}^{\prime }}$ and $v_{\mathrm{cls}}^{{\left(i\right)}^{\prime }}$ are the reconstructed audio and visual latent embeddings, respectively. Then, we constrain the reconstructed one and original features using the mean square error (MSE) loss

(17) $$L_{\mathrm{cross}\text{-}\mathrm{mod}}={∥a_{\mathrm{cls}}^{{\left(i\right)}^{\prime }}-a_{\mathrm{cls}}^{\left(i\right)}∥}_{2}^{2}+{∥v_{\mathrm{cls}}^{{\left(i\right)}^{\prime }}-v_{\mathrm{cls}}^{\left(i\right)}∥}_{2}^{2}.$$

By reconstructing latent-level summaries from different layers, the model develops a profound understanding that goes beyond shallow correlations. It learns to precisely align events in time and map their underlying semantic attributes, thus achieving the deep and robust cross-modal relations necessary for complex audio–visual learning.

### 4.5. Loss Functions

In various audio–visual downstream tasks, the employed training loss functions are diverse. We define the task-specific loss function as $L_{T}$ (e.g., Cross-Entropy Loss for AVE). The constraint item, $L_{\mathrm{cross}\text{-}\mathrm{mod}}$, is appended with hyperparameters α. The final loss $L_{F}$ is defined as:

(18) $$L_{F}=L_{T}+\alpha L_{\mathrm{cross}\text{-}\mathrm{mod}}$$

## 5. Experiments

### 5.1. Datasets

We evaluate our method on four publicly available audio–visual datasets covering three downstream tasks, including audio–visual event localization, audio–visual segmentation, and audio–visual question answering. The statistics of all datasets are summarized in Table 2.

**AVE** is an audio–visual event localization dataset originating from AudioSet [43]. It contains 4143 videos spanning 28 event categories. Each video lasts 10 s and is annotated at the segment level with event category labels.

**VGGSound-AVEL100k** is another audio–visual event localization dataset originating from VGGSound, comprising 101,072 video clips from 141 sound categories.

**AVSBench** is designed for audio–visual segmentation and includes two settings: Single Sound Source Segmentation (S4) with 4932 videos and Multiple Sound Source Segmentation (MS3) with 424 videos, covering 23 sound categories in total. Each video has a duration of 5 s and is annotated with pixel-level segmentation masks.

**MUSIC-AVQA** is an audio–visual question answering dataset consisting of 9288 videos and 45,867 question–answer pairs. It contains 33 question templates and 42 candidate answers, requiring joint reasoning over audio and visual modalities.

### 5.2. Downstream Tasks and Evaluation Protocols

**Audio–Visual Event Localization (AVE)** focuses on localizing audio–visual events that occur simultaneously in both audio and visual streams within a video. We implement the comparison experiments on the AVE dataset [42] and VGGSound-AVEL100k [29]. Following LAVISH, we extract audio and visual features with shared backbones, then we concatenate the audio and visual features and attach a linear layer to obtain the final audio–visual event prediction. The fraction of correctly predicted segments is regarded as the evaluation metric.

**Audio–Visual Segmentation (AVS)** aims to segment sound-emitting objects or regions in a video at the pixel level. We evaluate on AVSBench [22], which contains two settings: (1) Single Sound Source Segmentation (AVSBench-S4); (2) Multiple Sound Source Segmentation (AVSBench-MS3). We combine our adapter-based feature extractor with the original AVS model. The mean Intersection-over-Union (mIoU) of the predicted segmentation and the ground truth masks is used as an evaluation metric.

**Audio–Visual Question Answering (AVQA)** requires answering questions by integrating audio and visual information from a video. Experiments are conducted on the MUSIC-AVQA dataset [25], with a pre-trained text encoder employed to extract question features. For evaluation, we adopt accuracy as the primary metric, calculated by comparing model predictions with ground-truth answers.

### 5.3. Implementation Details

For a fair comparison, we preprocess the input video following the approach used in LAVISH, DG-SCT, and CoPL. Specifically, we sample one frame per second from the video for all downstream tasks. $T_{k}$ is set to 0.15 for all experiments. α is set to 0.0006 for AVE and AVQA tasks and 0.001 for AVS. Values of $T_{k}$ and α are compared in the ablation study. In terms of architecture, the joint spatial–temporal modeling interaction is integrated into the penultimate encoder layer and is applied only once, whereas the cross-modal latent reconstruction is performed after each encoder block. We set the down-sampling ratio of the adapter to 8 for the AVE task and 4 for the other tasks.During training, we employ the Adam optimizer with its default hyperparameters. The learning rate is set to 1 × 10^−4^ for the cross-modality reconstruction module. For the trainable adapter parameters in the downstream networks, the learning rates are set to 3 × 10^−5^ for ViT-based models and 6 × 10^−5^ for Swin-based models across all tasks. Notably, the input image resolution is configured as 224 × 224 for ViT backbones and 192 × 192 for Swin backbones. The batch size is set to 2 for AVE, 4 for AVS, and 1 for AVQA. All experiments were conducted on an NVIDIA RTX 3090 GPU using PyTorch 1.7.1. For additional details, please refer to Appendix A.

### 5.4. Results and Analysis

**Audio–Visual Events Localization.** The compared methods include models that solely train downstream networks (e.g., CMBS, VSCG, AVEL) and the PETL approach based on pre-trained transformers (e.g., LAVISH, DG-SCT, STG-CMA, and CoPL). For a fair comparison, we maintain the downstream network as a classification layer in all fine-tuning paradigms. Results in Table 3 show that our method achieves superior performance across the board. Specifically, with a ViT-L encoder, our approach surpasses LAVISH by 2.7 points and outperforms CoPL by 1.6 points, while with Swin-L encoders, our model outperforms STG-CMA by 1.8. Notably, LAVISH introduces 13.5 M additional parameters, while STG-CMA and DG-SCT require 19 M and 187 M tunable parameters; in contrast, JMSC maintains high efficiency, adding only 8.2 M parameters when using Swin-L encoders. To further evaluate the generalizability of our approach, we conduct experiments on the VGGSound-AVEL100k benchmark. As reported in Table 4, JMSC achieves state-of-the-art results, reaching 63.7% accuracy with the ViT-L encoder and 65.3% with the Swin-V2-L encoder. Moreover, Swin-based encoders consistently outperform ViT-based counterparts across all tasks. We attribute this to Swin’s ability to capture multi-scale visual representations, making it more robust to object size variations and spatial shifts caused by camera movement.

**Audio–Visual Segmentation.** Evaluation results for the Audio-Visual Segmentation task are summarized in Table 5. Compared with the PETL method, our approach demonstrates substantial performance improvements and outperforms both LAVISH and CoPL by 4.5 and 2.0 points, respectively, on the AVSBench-S4 dataset when using the ViT-L encoder. Notably, our method achieves superior performance with only 9.7 M additional parameters when employing the Swin-V2-L encoder compared with AVMoE and DG-SCT. Furthermore, when compared with AVSBG—a network specifically tailored for AVS tasks, our method surpasses it by 1.9 points on the challenging AVSBench-MS3 dataset. These results underscore the superior capability of our model in handling complex multimodal scenes.

**Audio–Visual Question Answering.** Finally, we conducted a comparative analysis with existing methods on the MUSIC-AVQA dataset, as shown in Table 6. Compared with the PETL method, our model outperforms LAVISH by 2.2 on the audio question. Notably, our model demonstrates a significant improvement in the most challenging audio–visual question types, highlighting its ability to effectively integrate and understand multimodal information. Experiments across multiple audio–visual downstream tasks and diverse datasets validate the generalization, robustness, and effectiveness of our method in adapting pre-trained models for comprehensive audio–visual learning.

**Comprehensive Quantitative Analysis.** To further validate the robustness and superiority of JMSC, we conducted additional statistical and multi-metric evaluations. First, regarding statistical significance, we repeated the experiments on the AVE dataset using three different random seeds—0, 42, and 1234. Our method achieved a mean accuracy of **80.5%** with a standard deviation of ±**0.3%**. This narrow confidence interval, combined with a significant margin over the runner-up method (LAVISH, 78.1%), confirms that our improvements are statistically significant and not result from random variations.

Second, regarding evaluation metrics, while Accuracy is the standard protocol for AVE and AVQA, the AVS task benefits from boundary-sensitive metrics. In addition to mIoU, we evaluated the F1-score for the AVS task. JMSC achieves an F1-score of 89.6 on AVSBench-S4 and 68.2 on AVSBench-MS3, consistently outperforming the baseline methods. This indicates that our CMLR and JSTM module not only improves region overlap but also enhances the precision of object boundary delineation.

### 5.5. Ablation Study

**Analysis of the Proposed Modules.** In this section, we perform a detailed ablation study to assess the individual contributions of the CMLR and JSTM modules, using ViT-L as the shared encoder in all ablation experiments. As shown in Table 7, the results highlight the significant impact of both modules on overall performance. CMLR yields notable improvements in AVS and AVQA tasks, while JSTM further enhances performance in AVE and AVEL. The highest performance is achieved when both modules are combined. These findings underscore the complementary roles of semantic reconstruction and spatial–temporal modeling. These results not only validate the effectiveness of the modules but also confirm the underlying mechanisms: the performance gain from CMLR indicates successful cross-modal semantic completion, while the improvement from JSTM demonstrates that coupling spatial and temporal dimensions is essential for consistent object tracking.

**Effectiveness of Cross-Modal Latent Reconstruction.** We analyzed the interaction between contrastive learning and cross-modal latent reconstruction on the AVE and AVS tasks. The contrastive learning setting employs the memory bank mechanism [55]. As shown in Table 8, cross-modal latent reconstruction achieves better performance gains than contrastive learning. Notably, using cross-modal latent reconstruction alone achieves results compared with the combined approach. This can be attributed to the fact that when deep semantic information across modalities can be mutually reconstructed and comprehended, it naturally fosters alignment, thereby reducing the necessity for explicit contrastive learning.

**Figure 5:**
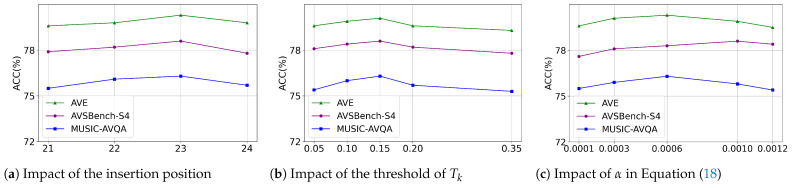
Ablation study results, where the y-axis in all subfigures represents accuracy. (**a**) shows the impact of the insertion position of our joint spatial–temporal modeling module, with the x-axis indicating the encoder layer index. (**b**) illustrates the effect of the relevance threshold in Equation (7) on performance. (**c**) presents the influence of the hyperparameter α, with the x-axis in both (**b**,**c**) representing the parameter values.

**The Insertion Position of the Joint Spatial–Temporal Modeling Module.** In this section, we evaluate the impact of the insertion position of the joint spatial–temporal modeling module. The experiments are conducted on the AVE and AVS tasks, with the insertion position ranging from the last layer to the fourth-to-last layer (21–24). The results shown in Figure 5a indicate that inserting the module at the −2 layer yields the best performance. As the module is inserted deeper into the network, it captures increasingly higher-level semantic features. However, this also introduces more complex information, requiring additional layers to integrate it effectively. This suggests that the depth of interaction plays a crucial role in optimizing performance.

**Impact of the Hyperparameter $T_{k}$.** We evaluate the influence of the threshold $T_{k}$ in Equation (7), which controls the selection of audio-related visual regions. As shown in Figure 5b, setting $T_{k}$ to 0.15 yields the best performance. As $T_{k}$ increases, the results initially improve due to the suppression of irrelevant regions. However, excessively high values of $T_{k}$ result in overly strict filtering, which limits the model’s ability to capture the useful extent of audio–visual correspondences, thereby hindering effective spatial extraction.

**Impact of the Hyperparameter α.** We conduct an ablation study on α in Equation (18), with the results shown in Figure 5c. It demonstrates that an appropriate consistency constraint loss is critical for optimal performance. Specifically, excessively large constraints would lead the model to overly focus on reconstruction during training, while overly small constraints have a negligible impact. Additionally, the goal of latent reconstruction is similar to the role of the loss in segmentation tasks. Therefore, on the AVS task, a larger value of α leads to relatively less performance degradation.

**Fusion Strategies in the Joint Spatial–Temporal Modeling Module.** This part of the study explores how different spatial–temporal fusion methods affect performance. We proposed three fusion strategies, as shown in Table 9. The optimal results are obtained when temporal features are element-wise added to spatial features. In addition, when temporal features are integrated into the CLS token of the spatial features, there is a slight drop in performance. This can be attributed to the fact that direct addition enables self-attention among patches, which facilitates more effective integration of spatial–temporal features. In contrast, the attention-based fusion method performs the worst. This may be due to excessive attention computations within the process, which disrupt the intrinsic relationships between the spatial and temporal features, ultimately leading to suboptimal results.

**Number of Joint Spatial–temporal Modeling Modules.** This section investigates the impact of repeatedly using the joint spatial–temporal modeling module. Starting from the penultimate layer and continuing downwards, the results are illustrated in Table 10, showing that excessive usage leads to increased redundant information, resulting in worse performance. To optimize both efficiency and results, this module is executed only once.

**Efficiency Analysis.** We evaluate the efficiency of the model by analyzing its additional parameters, inference speed, and memory usage. Both LAVISH and our model utilize the shared Swin-V2-L as the backbone, while DG-SCT utilizes Swin-V2-L for visual encoding and HT-SAT for audio encoding. Experiments are conducted on the AVE dataset, with the batch size set to 2, and the results are presented in Table 11. Our model delivers the best performance while requiring minimal training memory and parameter overhead, and it boasts the fastest inference speed. This demonstrates the efficiency of the proposed JMSC model.

**Analysis of Design Granularity.** We initially hypothesized that capturing multi-scale details would lead to finer-grained representations. Therefore, we explored two theoretically comprehensive variants of the JSTM:

- Hierarchical Temporal Aggregation: Instead of a single global pooling, we fused temporal features at multiple scales (e.g., aggregating contexts every 3, 5, and 7 s) to capture both short-term and long-term semantics.
- Multi-granularity Spatial Gating: Rather than relying on a single optimal threshold, we integrated visual regions activated by varying relevance thresholds (e.g., fusing features from $T_{k}\in \left\{0.1,0.25,0.5\right\}$) to capture sounding objects at different salience levels.

As shown in Table 12, contrary to intuitive expectations, these complex designs did not bring performance gains and even caused degradation (e.g., a 0.9% drop on AVE). We attribute this to the specific nature of the PETL regime. Since the pre-trained backbone is frozen, the learnable capacity is concentrated in the lightweight adapters. Introducing heavy feature aggregation or multi-scale redundancy tends to lead to overfitting on the downstream datasets. Furthermore, excessive fusion may disrupt the semantic distribution of the original pre-trained features. Consequently, our ‘Spatial-Fine + Temporal-Global’ design proves to be the optimal trade-off between efficiency and robustness.

**Impact of Audio Modality.** To explicitly quantify the contribution of auditory information, we conducted an experiment comparing our full audio–visual framework against a uni-modal visual baseline, using Swin-V2-L as the encoders. In the uni-modal setting, we removed the audio branch and relied solely on the visual encoder with adapters. The results are summarized in Table 13.

As observed, the exclusion of audio cues leads to a significant performance degradation across all datasets. Specifically, the performance drops by 8.1% on AVE and 9.8% on AVSBench-S4. The most substantial decline occurs in the MUSIC-AVQA dataset, where accuracy falls by 13.7% (from 77.3% to 63.6%). These results confirm that our JMSC framework effectively leverages complementary audio–visual semantics rather than overfitting to visual features, thereby validating the contribution of audio modality.

**Systematic Analysis of Key Components.** To further substantiate the design choices of JMSC, we conducted a comprehensive systematic analysis on the AVE dataset. We investigated the individual contributions of different masking strategies, the impact of spatial filtering, and the effect of reconstruction depth. The results are reported in Table 14. Analysis are shown as follows:

- Masking Strategy: As shown in Table 14(a), employing both channel and temporal masking yields the best performance. Channel masking encourages the model to infer semantic attributes, while temporal masking enforces context-aware prediction. Combining them provides a complementary supervisory signal.
- Spatial Filtering: Table 14(b) demonstrates that removing the spatial filtering mechanism (i.e., treating all visual regions equally) leads to a performance drop. This confirms that explicitly filtering out visually irrelevant regions based on audio cues $T_{k}$ is essential for precise localization.
- Reconstruction Depth: Regarding the number of reconstruction layers in Table 14(c), we observe that applying CMLR to all encoder layers outperforms applying it only to the top layers. This suggests that bridging the modality gap requires alignment across the entire hierarchical feature space, not just at the semantic bottleneck.

### 5.6. Limitations and Future Work

We observe that our method may struggle with scenarios involving extremely small or thin sounding objects, such as the fine strings of a violin. Although the proposed JSTM module can correctly localize the coarse region of the sound source, precise boundary delineation remains challenging. This limitation mainly arises from the spatial resolution bottleneck inherent in parameter-efficient transfer learning with frozen visual backbones. Specifically, the fixed patch size (e.g., 16×16) restricts the model’s ability to capture fine-grained spatial structures, leading to accurate localization but imprecise segmentation in AVS tasks. To address this resolution-efficiency trade-off, future work could explore a “Coarse-to-Fine” input adaptation strategy. Instead of altering the frozen backbone, one could pre-process the visual input based on cross-modal relevance. For instance, in AVQA tasks, we could compute the cosine similarity between the question text (or audio query) and visual patches to identify a Region of Interest (RoI). By cropping and resizing this high-relevance region to the standard input size, we can effectively increase the relative resolution of small sounding objects without increasing the computational budget. Integrating such dynamic input mechanisms with our JMSC framework offers a promising path toward more robust fine-grained audio–visual understanding.

### 5.7. Qualitative Analysis

**Figure 6:**
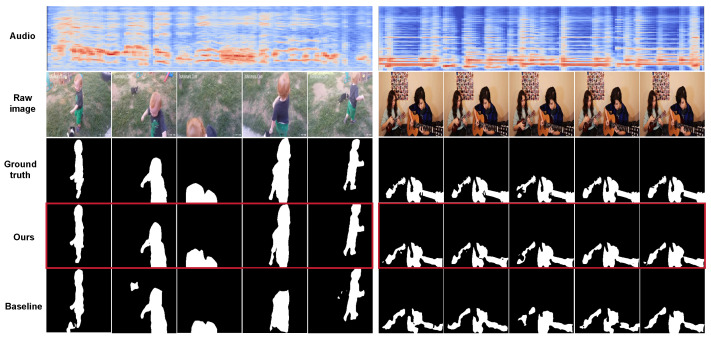
Qualitative example comparing LAVISH and our JMSC framework on the AVS task.

**Figure 7:**
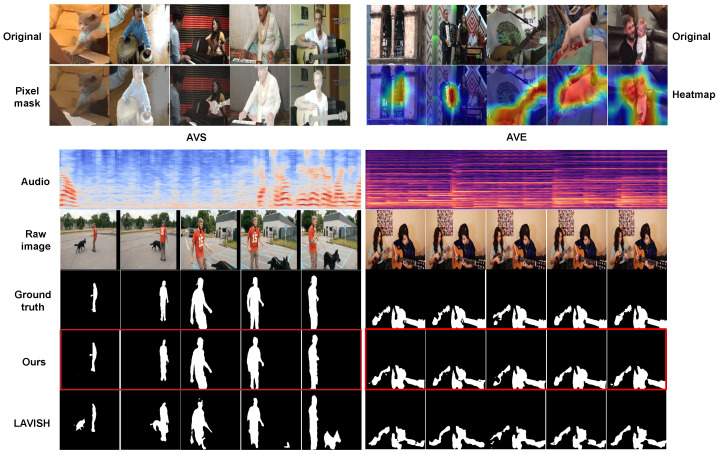
More qualitative examples on the AVSBench and AVE dataset.

We present qualitative visualizations from the AVS and AVE tasks to further demonstrate the effectiveness of our proposed method. Figure 6 presents qualitative results on the AVS task. As shown in the figure, our model produces more precise object boundaries compared with the baseline method LAVISH. In the left example, our method correctly segments the speaking child while suppressing the nearby silent dog, whereas LAVISH incorrectly highlights the dog. In the right example, our model accurately delineates partially occluded instruments such as the violin and piano, demonstrating strong robustness in complex scenes. Figure 7 provides a joint qualitative analysis of the AVE and AVS tasks. Specifically, the left of the first two columns in Figure 7 illustrates the model’s ability to accurately segment sound-producing objects, precisely outlining their shapes in complex scenes. For example, it successfully localizes fine-grained sound sources such as guitar and keyboards. The right two rows show the model’s capacity to focus on critical visual regions associated with various AVE events, as visualized using Grad-CAM. The lower part of Figure 7 presents qualitative segmentation results for the AVS task, where our method achieves more accurate object localization and clearer boundary delineation compared with LAVISH. Overall, these qualitative results across both AVE and AVS tasks highlight the strong interpretability and generalization ability of our model.

**Figure 8:**
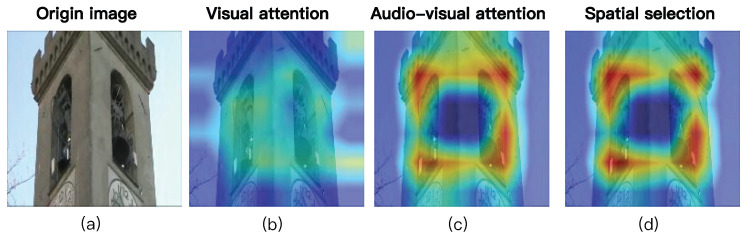
Spatial selection mechanism: interpretability visualization. (**a**) Visual saliency: the model attends to all potential objects. (**b**) Raw audio–visual attention: audio queries localize the sound source region but retain background noise. (**c**,**d**) Ours (Spatial selection): attention map refined via spatial filtering threshold $T_{k}$, effectively suppressing irrelevant regions and focusing on the actual sounding object, verifying JMSC’s spatial selection capability.

**Interpretability of Spatial Selection.** To substantiate the role of sound in our framework, we visualize the attention evolution on the AVE dataset in Figure 8. Column (a) displays the original image for reference. Column (b) shows that relying solely on visual features results in ambiguous attention, which incorrectly focuses on the silent tower (i.e., the tower not associated with the sound source). Column (c) illustrates that introducing audio cues helps roughly localize the sound source, yet the attention heatmap remains coarse and still contains irrelevant noise. In contrast, Column (d) presents the final heatmap adjusted by our spatial selection mechanism. By filtering out regions below the relevance threshold ($T_{k}$), the model successfully further reduces noise—specifically, the irrelevant regions in the middle and lower parts of the tower—suppresses silent objects, and achieves precise sound source localization. This confirms that JMSC explicitly leverages sound significance to modulate spatial attention effectively.

## 6. Conclusions

In this work, we proposed JMSC, a novel framework that adapts unimodal models pre-trained solely on natural images for audio–visual learning. By introducing cross-modal latent reconstruction, JMSC enhances the model’s capacity to capture the intrinsic semantic correlations and temporal consistency across modalities. Additionally, we introduce joint spatial–temporal modeling, which effectively models the co-changes of spatial and temporal features, resulting in enhanced spatial–temporal representation. Extensive experiments on four widely used datasets covering five experimental settings demonstrate the superiority and robustness of our approach. Furthermore, ablation studies confirm the effectiveness of each proposed component. In future work, we plan to further investigate the generalization ability of JMSC in more diverse and complex audio–visual scenarios, and explore visual region-of-interest extraction to improve the model’s performance on small sounding objects.

## Figures and Tables

**Table 1:** Summary of main notations and symbols used in this paper, where CLS is the abbreviation for Classification.

Symbol	Description
$v_{t}$	Visual token embeddings at time *t*
$a_{t}$	Audio token embeddings at time *t*
$v_{cls}^{t}$	CLS token of the visual modality at time *t*
$a_{cls}^{t}$	CLS token of the audio modality at time *t*
$v_{p}^{t}$	Visual patch features at time *t*
$\Theta_{a}^{s},\Theta_{v}^{s}$	Linear projection layers for audio and visual features
⊙	Element-wise multiplication
$M_{sa}^{t}$	Audio-guided spatial attention map at time *t*
$T_{k}$	Threshold for spatial region filtering
β	Learnable fusion coefficient for spatial–temporal integration
$v_{global}^{\prime }$	Temporally pooled global visual representation
F(·)	Linear projection layers for feature fusion
$V_{t}^{f}$	Final fused spatial–temporal feature at time *t*
$M_{T}$	Temporal masking matrix in latent space
$M_{C}$	Channel masking matrix in latent space
$x_{cls}^{\left(i\right)}$	CLS token at the *i*-th encoder layer (x∈{a,v})
$x_{cls}^{\left(i\right)}\left[\mathrm{mask}\right]$	Masked CLS token at the *i*-th layer
$a_{cls}^{\prime (i)}$	Reconstructed audio CLS token
$v_{cls}^{\prime (i)}$	Reconstructed visual CLS token
$L_{cross\text{-}mod}$	Cross-modal latent reconstruction loss

**Table 2:** **Summary of datasets.** This table reports key statistics of each benchmark, including the total number of videos, annotated frames, category or answer space, and supervision types. The supervision format includes class labels, pixel-level masks, and question–answer pairs. The statistics are compiled based on the official descriptions of the respective datasets and prior studies [13].

Datasets	Samples	Frames	Classes/ Answers	Modality	Supervision
AVE [42]	4143	41,430	28	video	class-level
AVSBench [22]	5356	12,972	23	video	pixel-level
VGGSound-AVEL100k [29]	101,072	1,010,720	141	video	class-level
MUSIC-AVQA [25]	9288	45,867	42	video	QA-pairs

**Table 3:** **Results on Audio–Visual Events Localization.** We compare our proposed JMSC with previous methods on the AVE dataset. * denotes models trained with random data augmentation [44]. Our approach achieves optimal results by employing shared encoders. Bold indicates the best performance.

Method	Visual Encoder	Audio Encoder	Additional Params (M)	Total Params (M)	Acc
AVEL [42]	Resnet-152	VGGish	3.7	136.0	74.0
CMRAN [27]	Resnet-152	VGGish	15.9	148.2	78.3
CMBS [28]	Resnet-152	VGGish	14.4	216.7	79.7
PSP [20]	VGG19	VGGish	1.7	217.4	77.8
VSCG [45]	VGG19	VGGish	N/A	N/A	79.7
DG-SCT [13]	Swin-V2-L	HTS-AT	187.1	448.4	78.3
CCLN [46]	VGG-19	VGG-like	N/A	N/A	79.9
AVCSF [47]	VGG-like	Resnet-152	N/A	N/A	80.6
LAVISH [38]	ViT-L-16 (shared)		13.5	340.7	78.1
CoPL [14]	ViT-L-16 (shared)		5.0	332.8	79.2
**Ours**	ViT-L-16 (shared)		11.9	337.5	**80.8**
LAVISH [38]	Swin-V2-L (shared)		8.5	238.8	81.1
CoPL [14]	Swin-V2-L (shared)		1.7	232.3	81.9
AVMoE [48]	Swin-V2-L (shared)		147.5	374.4	81.5
**Ours**	Swin-V2-L (shared)		8.2	236.2	**83.2**
CG-MRLN [49] *	Swin-V2-L	CNN14	N/A	299.4	83.5
STG-CMA [17] *	Swin-V2-L (shared)		19.0	254.0	82.5
Ours *	Swin-V2-L (shared)		8.2	236.2	**84.3**

**Table 4:** **Results on VGGSound-AVEL100k.** We compare our proposed JMSC with previous audio–visual event localization methods.

Method	Visual Encoder	Audio Encoder	Additional Params (M)	Total Params (M)	Acc
AVEL [42]	VGG-19	VGGish	N/A	136.0	55.7
CMBS [28]	Swin-V2-L	VGGish	30.6	216.7	57.1
CPSP [29]	VGG-19	VGGish	N/A	N/A	59.9
CG-MRLN [49]	Swin-V2-L	CNN14	N/A	299.4	64.1
FG-MRLN [49]	Resnet-101	CNN14	N/A	346.5	64.3
CMRAN [27]	Swin-V2-L	CNN14	15.9	148.2	59.9
PSP [20]	VGG-19	VGGish	1.7	217.4	58.3
**Ours**	ViT-L-16 (shared)		13.5	337.5	63.7
**Ours**	Swin-V2-L (shared)		8.2	236.2	**65.3**

**Table 5:** **Results on Audio–Visual Segmentation.** We evaluate our method on the AVSBench-S4 and AVSBench-MS3 datasets (shown as S4 and MS3 in the table) using the mIoU metric. Our approach achieves the best results on both datasets.

Method	Visual Encoder	Audio Encoder	Additional Params (M)	Total Params (M)	Setting	
					S4	MS3
					mIoU	mIoU
AVS [22]	PVT-v2	VGGish	82.3	174.5	78.7	54.0
LGVT [50]	PVT-v2	VGGish	N/A	N/A	74.0	40.7
CATR [51]	Resnet-50	VGGish	N/A	N/A	74.8	52.8
Diff-AVS [52]	Resnet-50	VGGish	N/A	N/A	75.8	49.7
AVSBG [1]	PVT-v2	VGGish	85.5	N/A	81.7	55.1
MoCA [53]	XCiT	ViT-L-16	1.3	297.1	68.0	57.0
DG-SCT [13]	Swin-V2-L	HTS-AT	196.6	521.4	80.9	53.5
AVMoE [48]	Swin-V2-L	HTS-AT	271.6	501.2	81.1	54.5
LAVISH [38]	ViT-L-16 (shared)		27.1	375.5	74.1	49.1
CoPL [14]	ViT-L-16 (shared)		4.7	353.4	76.6	—
**Ours**	ViT-L-16 (shared)		12.8	361.8	**78.6**	**54.3**
LAVISH [38]	Swin-V2-L (shared)		18.3	266.4	80.1	49.8
CoPL [14]	Swin-V2-L (shared)		2.8	253.0	80.7	—
**Ours**	Swin-V2-L (shared)		9.7	261.3	**82.1**	**57.0**

**Table 6:** **Results on Audio–Visual Question Answering.** We report accuracy on three types of questions, e.g., audio (AQ), visual (VQ), and audio–visual (AVQ) on the MUSIC-AVQA dataset.

Method	Visual Encoder	Audio Encoder	Additional Params (M)	AQ	VQ	AVQ	Avg
AVQA [25]	SwinV2-L	VGGish	12.2	75.5	75.6	74.5	72.2
Pano [26]	Faster RCNN	VGGish	N/A	70.7	72.6	66.6	68.9
PSTP [9]	CLIP-ViT-B	VGGish	4.3	70.9	77.3	72.6	73.6
AVMoE [48]	SwinV2-L	HTS-AT	4.3	77.6	82.7	71.9	75.7
DG-SCT [13]	Swin-V2-L	HTS-AT	186.3	77.4	81.9	70.7	74.8
LAVISH [38]	ViT-L-16 (shared)		21.1	74.1	73.6	74.7	74.4
QA-TIGER [54]	Clip-ViT-L	VGGish	N/A	78.5	85.1	73.4	77.6
AVAF-Net [16]	Clip-ViT-L	VGGish	N/A	78.1	82.3	72.1	75.9
**Ours**	Clip-ViT-L (shared)		14.8	78.3	85.6	73.9	**77.9**
LAVISH [38]	Swin-V2-L (shared)		21.1	75.7	80.4	70.4	74.0
STG-CMA [17]	Swin-V2-L (shared)		43.1	78.7	83.0	72.3	76.2
CoPL [14]	Swin-V2-L (shared)		1.8	77.3	77.6	76.3	76.7
**Ours**	Swin-V2-L (shared)		8.7	77.9	78.1	77.5	**77.3**

**Table 7:** Ablation study of the proposed module. Bold indicates the best performance.

Module		AVE		AVS		AVQA
CMLR	JSTM	AVE Acc	AVEL Acc	S4 mIoU	MS3 mIoU	Avg
		76.9	59.6	76.6	49.7	73.6
✓		78.7	60.5	78.1	52.3	75.4
	✓	80.1	61.2	77.6	51.4	75.3
✓	✓	**80.8**	**63.7**	**78.6**	**54.3**	**76.3**

**Table 8:** Ablation study on the effectiveness of CMLR module. “Align” denote contrastive learning.

Method		AVE	AVS	
Align	CMLR	Acc	S4 mIoU	MS3 mIoU
		76.9	76.6	49.7
✓		77.6	77.8	50.5
	✓	**78.7**	78.0	**52.3**
✓	✓	78.7	**78.1**	52.3

**Table 9:** Results of different methods of joint spatial–temporal feature fusion on all datasets. Add denotes element-wise summation; Attention denotes fusing the temporal and spatial features after calculating attention; Add2Cls means adding the temporal features to the spatial features’ CLS token. Results show that simple add is the best.

Module	AVE		AVS		AVQA
Method	AVE	AVEL	S4 mIoU	MS3 mIoU	Avg
Add	**80.8**	**63.7**	**78.6**	**54.3**	**76.3**
Add2CLS	79.7	61.9	78.4	54.0	75.8
Attention	78.3	60.1	77.1	53.3	75.3

**Table 10:** Impact of the number of JSTM modules.

Dataset	Use Once	Use Twice	Use Third
AVE	**80.8**	79.1	78.7
AVSBench-S4	**78.6**	77.4	77.1
MUSIC-AVQA	**76.3**	75.5	75.1

**Table 11:** Efficiency Analysis: Comparison of additional parameters, inference speed, and memory usage on the AVE dataset.

Method	Additional Params (M)	Training Memory (GB)	Samples per Second	Acc
LAVISH	8.5	18.7	1.40	81.1
DG-SCT	187.1	22.6	1.24	78.3
**Ours**	**8.2**	**17.9**	**1.62**	**83.2**

**Table 12:** Ablation study on the design granularity of joint spatial–temporal modeling. We compare our approach against more complex method variants. “H” denotes “Hierarchical Temporal” (a component that aggregates temporal features to capture both short-term and long-term semantic patterns). “M” refers to “Multi-granularity Spatial” (a module designed to capture varying salience levels of audio-correlated objects).

Module	AVE		AVS		AVQA
Method	AVE	AVEL	S4 mIoU	MS3 mIoU	Avg
**Ours**	**80.8**	**63.7**	**78.6**	**54.3**	**76.3**
M + H	79.9	63.2	78.1	53.7	75.6

**Table 13:** Impact of audio modality. We compare the performance of using only the visual modality versus our full audio–visual framework across three datasets.

Dataset	Input Modality	Metric	Result
AVE	Visual Audio + Visual	Accuracy	75.1 **83.2**
AVSBench-S4	Visual Audio + Visual	mIoU	72.3 **82.1**
MUSIC-AVQA	Visual Audio + Visual	Accuracy	63.6 **77.3**

**Table 14:** Systematic ablation analysis on the AVE dataset. We evaluate (a) the impact of different masking strategies in CMLR, (b) the necessity of spatial filtering in JSTM, and (c) the effect of applying CMLR at different encoder depths.

Aspect	Variant	Accuracy (%)
(a) Masking Strategy (CMLR)		
	Channel Masking Only	78.1
	Temporal Masking Only	78.2
	**Channel + Temporal (Ours)**	**78.7**
(b) Spatial Filtering		
	w/o Spatial Filtering (No $T_{k}$)	79.6
	**w/Spatial Filtering (Ours)**	**80.1**
(c) Number of Reconstruction Layers		
	Last 1 Layer	77.6
	Last 6 Layers	78.4
	**All Layers (Ours)**	**78.7**

## Data Availability

Publicly available datasets were analyzed in this study. These data can be found here: [22,25,29,42].

## Appendix A. Hyperparameter Settings

To support reproducibility, we provide the detailed hyperparameter configurations and the tuning protocol used in our experiments. The final selected values for each task are summarized in Table A1.

- Fixed Parameters: Certain hyperparameters were fixed based on established literature or pre-trained model specifications to avoid unnecessary computational overhead. Specifically, the masking ratio for the CMLR module was fixed at 0.75, following the standard practice in Masked Autoencoders, where a 75% masking ratio is empirically validated as optimal for visual representation learning. Similarly, the input image resolutions (224×224 for ViT, 192×192 for Swin) were determined by the requirements of the pre-trained ImageNet backbones. In addition, the batch size, the down-sampling ratio of the adapter, and the learning rate for the adapter modules were set in accordance with standard practices in prior audio–visual learning works.
- Selected Parameters: For task-specific hyperparameters that are less standardized in the literature, including the spatial filtering threshold ($T_{k}$) and the consistency loss weight (α), we employed a grid search strategy on the validation set of each dataset. For instance, we searched α within {0.0001,0.0003,0.0006,0.001,0.0012} and $T_{k}$ within {0.1,0.15,0.2,0.25}. The configurations yielding the best performance metrics (Accuracy for AVE/AVQA, mIoU for AVS) on the validation split were selected for the final reporting.

**Table A1:** Detailed hyperparameter settings for different downstream tasks.

Hyperparameter	AVE	AVS	AVQA
Optimizer	Adam	Adam	Adam
Learning Rate (ViT)	3 × 10^−5^	3 × 10^−5^	3 × 10^−5^
Learning Rate (Swin)	6 × 10^−5^	6 × 10^−5^	6 × 10^−5^
Batch Size	2	4	1
Downsample ratio	8	6	4
Masking Ratio (CMLR)	0.75	0.75	0.75
Spatial Threshold ($T_{k}$)	0.15	0.15	0.15
Loss Weight (α)	0.0006	0.001	0.0006
Input Resolution (ViT)	224×224	224×224	224×224
Input Resolution (Swin)	192×192	192×192	192×192
